# Identification of HGD and GSTZ1 as Biomarkers Involved Metabolic Reprogramming in Kidney Renal Clear Cell Carcinoma

**DOI:** 10.3390/ijms23094583

**Published:** 2022-04-21

**Authors:** Jiyan Wang, Hongkai Chang, Meng Su, Yaya Qiao, Huanran Sun, Yongshan Zhao, Shuai Zhang, Changliang Shan

**Affiliations:** 1 State Key Laboratory of Medicinal Chemical Biology, College of Pharmacy and Tianjin Key Laboratory of Molecular Drug Research, Nankai University, Tianjin 300350, China; wangjy@mail.nankai.edu.cn (J.W.); 2120201229@mail.nankai.edu.cn (H.C.); 2120191147@mail.nankai.edu.cn (Y.Q.); 2120201192@mail.nankai.edu.cn (H.S.); 2 School of Life Science and Bio-Pharmaceutics, Shenyang Pharmaceutical University, Shenyang 110016, China; sumeng0322@163.com (M.S.); zhaoys@syphu.edu.cn (Y.Z.); 3 School of Integrative Medicine, Tianjin University of Traditional Chinese Medicine, Tianjin 301617, China; 4 State Key Laboratory of Drug Research, Shanghai Institute of Materia Medica, Chinese Academy of Sciences, Shanghai 201203, China

**Keywords:** tyrosine metabolism, fumarate, metabolism reprogramming, renal cell carcinoma

## Abstract

Kidney renal clear cell carcinoma (KIRC) with poor prognosis is the main histological subtype of renal cell carcinoma, accounting for more than 80% of patients. Most patients are diagnosed at an advanced stage due to being asymptomatic early on. Advanced KIRC has an extremely poor prognosis due to its inherent resistance to radiotherapy and chemotherapy. Therefore, a comprehensive understanding of the molecular mechanisms of KIRC and the development of effective early diagnostic and therapeutic strategies is urgently needed. In this study, we aimed to identify the prognosis-related biomarker and analyzed its relationship with tumor progression. Metabolic changes are an important feature of kidney cancer, where the reduction of fumarate allows us to target the tyrosine metabolic pathway. The homogentisate 1,2-dioxygenase (HGD) and glutathione S-transferase zeta 1 (GSTZ1) related with prognosis of KIRC was identified through bioinformatics analysis based on The Cancer Genome Atlas (TCGA) databases. Mechanistically, we found that decreased HGD and GSTZ1 promote aerobic glycolysis in KIRC, coordinate the balance of amino acid metabolism and energy metabolism in tumor cells, and ultimately activate the tumor cell cycle and tumor progression. In summary, we identified the tyrosine metabolizing enzymes HGD and GSTZ1 as biomarkers of KIRC, which will further the understanding of the tumor metabolism profile, provide novel strategies and theoretical support for diagnosing and treating KIRC and as referential for future clinical research.

## 1. Introduction

Renal cell carcinoma (RCC) is a malignant tumor that originates from renal tubular epithelial cells, accounting for nearly 90% of renal malignancies [1]. RCC is not a single disease, but represents several distinct types of cancer mainly including kidney renal clear cell carcinoma (KIRC), kidney papillary cell carcinoma (KIRP), and kidney chromophobe (KICH), which have defining histologies and genetic alterations and follow different clinical courses and have different responses to therapy. KIRC is the most typical subtype of renal cell carcinoma. It is usually asymptomatic, and approximately 30% of patients are diagnosed in the advanced stage. Advanced KIRC has an extremely poor prognosis due to its inherent resistance to radiotherapy and chemotherapy [2]. In particular, early stage tumors have a significantly better disease-free survival after resection than tumors of advanced stage [3]. However, Staehler et al. found that KIRC is susceptible to robotic radiosurgery, in which oncological and functional results are comparable to open partial nephrectomy for early stage tumors [4]. Despite advances in treatment including immunotherapy, radiochemotherapy, and targeted therapy, there were no reliable biomarkers for early screening and prognosis judgment of KIRC, which limits the progress of treatment. Hence, the discovery of biomarker for diagnosis against KIRC remains an urgent task.

Metabolic reprogramming is the hallmark of cancer [5]. As early as 1924, Otto Warburg found that tumor cells tend to metabolize glucose into lactate for energy even under aerobic conditions, so it is also known as the Warburg effect or aerobic glycolysis [6,7]. Aerobic glycolysis has been reported to be grade-dependently up-regulated in RCC [8], implicating the involvement of glucose metabolism in renal cancer progression. In addition, KIRC also showed dysregulated oxidative phosphorylation (OXPHOS), tricarboxylic acid cycle (TCA cycle), and amino acid metabolism [8,9]. Furthermore, glucose levels are closely related to the malignancy of RCC [10]. These undoubtedly indicate that significant metabolic alterations occur in kidney cancer. More importantly, metabolite changes are closely related to renal cancer progression, which provides a theoretical basis for targeting metabolic changes to identify biomarkers. Changes in cellular metabolism contribute to the development and progression of tumors, and also renders tumors vulnerable to interventions. Currently, there are drugs targeting the PI3K/AKT/mTOR pathway (temsirolimus and everolimus) that increase disease-free progression and survival and prolong disease stability by inhibiting HIFα expression [11]. We want to identify new biomarkers through a deeper exploration of the metabolic reprogramming mechanisms that occur in RCC, as further understanding of the metabolic basis of kidney cancer will lead to the development of effective forms of therapy for this disease.

In this study, we first analyzed the relationship between tyrosine metabolism and RCC based on the reported results and TCGA database, and found that HGD and GSTZ1 were significantly down-regulated and were associated with poor prognosis of KIRC. Additionally, reduced HGD and GSTZ1 induced a global arrest of amino acid metabolism. Not only that, but consistent with previous studies, energy-generating pathways (glycolysis, TCA cycle, and OXPHOS) in renal cancer cells were inhibited due to the reduction of HGD and GSTZ1. This suggests that starting from fumarate, the end product of tyrosine metabolism affects a cascade of amino acid metabolism and energy metabolism. At the molecular level, we found that SLC2A1, LDHA, GOT1, and GOT2 were regulated by HGD and GSTZ1 to alter the glucose uptake and energy metabolism of renal cancer cells. Collectively, we analyzed data by bioinformatics methods to identify HGD and GSTZ1 as biomarkers of KIRC from a metabolic perspective, and their expression changes altered the energy-producing pathways of tumor cell growth and proliferation, affecting cell cycle and tumor progression, which will provide important help for the clinical diagnosis and treatment of renal cell carcinoma. We aim to identify biomarkers of KIRC to facilitate diagnosis with the goal of early detection and early treatment.

## 2. Results

### 2.1. HGD and GSTZ1 Were Down-Regulated and Associated with Prognosis in KIRC

**Figure 1:**
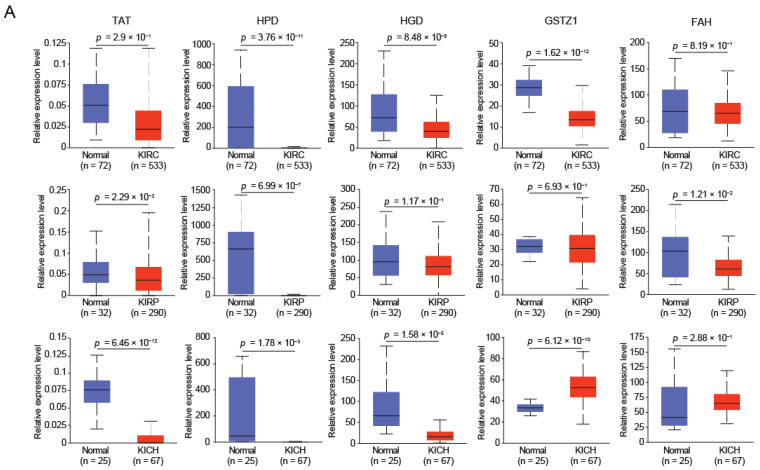

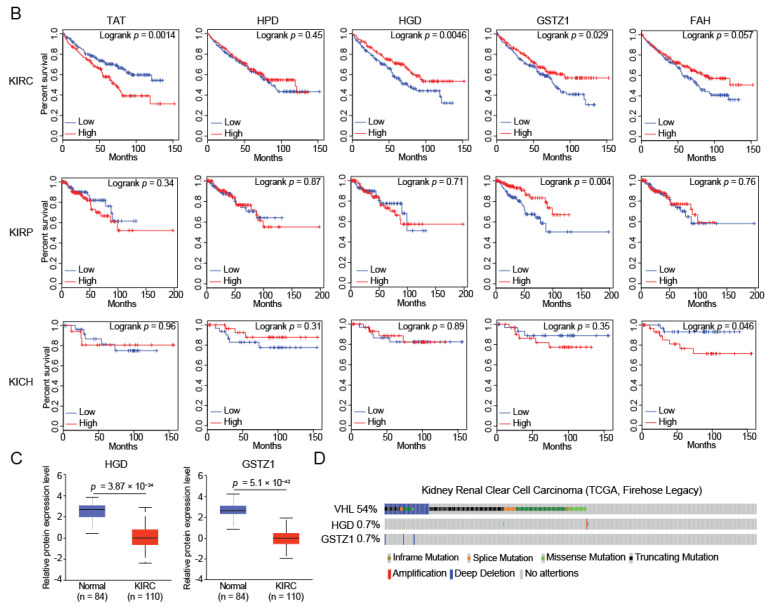
HGD and GSTZ1 are down-regulated and associated with prognosis in KIRC. (**A**) The tyrosine-metabolizing enzymes (TAT, HPD, HGD, GSTZ1, and FAH) mRNA expression levels between tumor and normal tissues in patients with RCC in TGCA database. (**B**) Overall survival of patients with RCC grouped by tyrosine-metabolizing enzymes’ expression through GEPIA web server. (**C**) The tyrosine-metabolizing enzymes (HGD and GSTZ1) protein expression levels between tumor and normal tissues in patients with KIRC in TGCA database. (**D**) The types and frequency of mutations of VHL, HGD and GSTZ1 in RCC patients.

Metabolic reprogramming is an important characteristic of tumorigenesis and development, and the reduction of fumarate has been reported to be significantly correlated with the occurrence of renal cancer [10]. Fumarate is not only mediated in the TCA cycle, but is also a product of tyrosine metabolism [12]. To this end, we analyzed the expression levels of tyrosine-metabolizing enzymes in three common types of renal cancer (KIRC, KIRP, and KICH), and found that the expression level of tyrosine-metabolizing enzymes was down-regulated in renal cancer compared with normal tissues (Figure 1A). We aimed to identify biomarkers used to indicate progression and prognosis in renal cancer. Therefore, we analyzed the prognostic correlation of tyrosine metabolizing enzymes in three renal cancers and found that TAT, HGD, and GSTZ1 were correlated with the prognosis of KIRC, while GSTZ1 was significantly correlated in KIRP (Figure 1B). In KICH, no significantly associated candidates were found. Considering expression differences and prognostic correlations together, we believe that HGD and GSTZ1 have the potential to be biomarkers of KIRC. In addition, we also found that the protein levels of HGD and GSTZ1 were also significantly down-regulated in KIRC (Figure 1C).

Von Hippel Lindau (VHL) is an inherited cancer syndrome in which affected individuals are at risk of the development of tumors in a number of organs, including the kidneys [13,14]. Mutations occurred in 54% of KIRC patients from the TCGA database, so genetic mutations in VHL are thought to be a driver of kidney carcinogenesis (Figure 1D). In parallel, mutations in VHL also induce the occurrence of other tumors, including cerebellar and spinal hemangioblastomas, retinal angiomas, endolymphatic sac tumors [15], pancreatic neuroendocrine tumors [16], and pheochromocytoma [17]. Therefore, VHL is not a specific biomarker for renal cancer. However, the mutation rate of HGD and GSTZ1 was only 0.7% in KIRC, indicating that the function of HGD and GSTZ1 in renal cancer depends on the expression abundance. In our previous study, it was found that the abundance of tyrosine metabolizing enzymes in kidney tissue was only lower than that in liver tissue [12], which indicated that active tyrosine metabolism maintained physiological activities in kidney tissue. In short, we found that HGD and GSTZ1 are down-regulated in KIRC and associated with poor prognosis, and have the potential to be biomarkers.

### 2.2. GOT1/2 Was Required for Amino Acid Metabolism Regulated by HGD/GSTZ1

**Figure 2:**
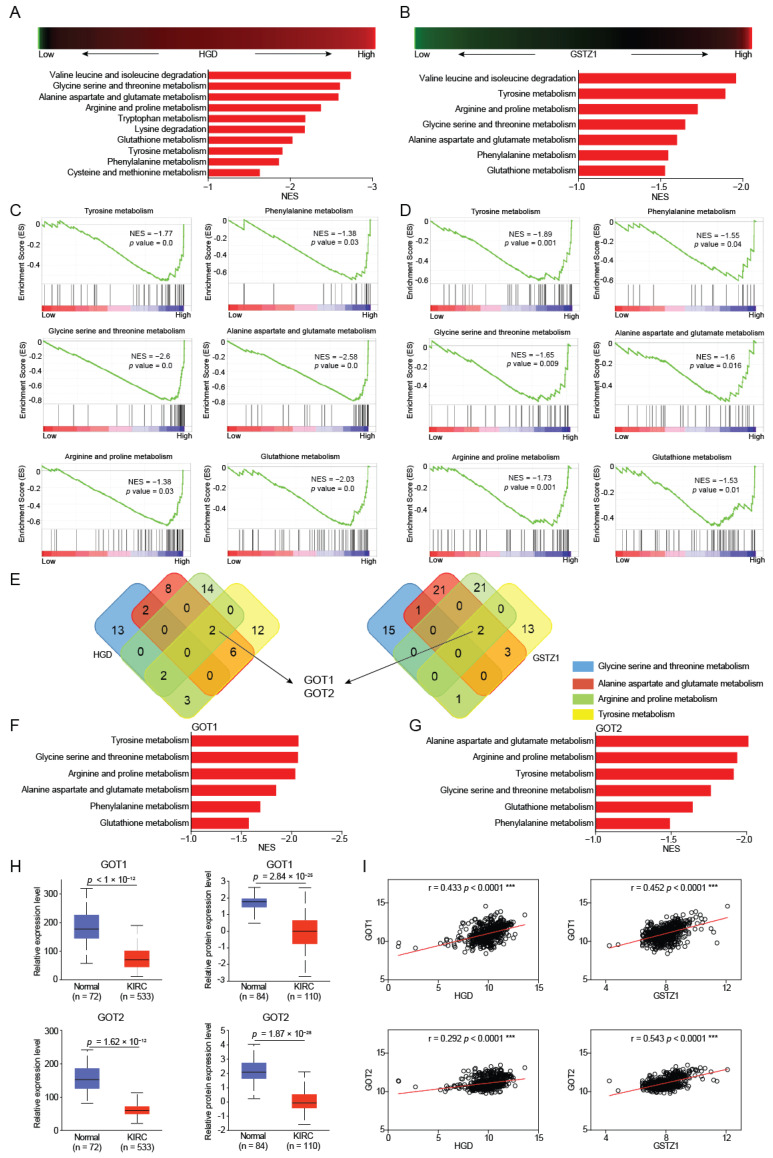
GOT1/2 mediates amino acid metabolism regulated by HGD/GSTZ1. (**A**,**B**) The core-enriched signaling pathways in high and low HGD or GSTZ1 groups. NES, normalized enrichment score. (**C**,**D**) GSEA pathway enrichment analyses of HGD or GSTZ1 signature in patients with RCC from the TCGA datasets. (**E**) The overlapping analysis for related genes of amino acid metabolism in RCC patients. (**F**,**G**) The core-enriched signaling pathways in high and low GOT1 or GOT2 groups. NES, normalized enrichment score. (**H**) The mRNA and protein expression levels of GOT1 and GOT2 between tumor and normal tissues in patients with KIRC in TGCA database. (**I**) The expression correlation between HGD/GSTZ1 and GOT1/GOT2. *** *p* < 0.001.

To uncover the functional mechanism of HGD and GSTZ1 in KIRC, we utilized the publicly available datasets (The Cancer Genome Atlas-Kidney Clear Cell Carcinoma) for pathway enrichment analysis. First, we divided the patients into two groups with a high and low expression of HGD or GSTZ1. We next sought to investigate the altered signaling pathways driven by the increase of HGD or GSTZ1 in KIRC. Gene set enrichment analysis (GSEA) of pathway enrichment showed that amino acid metabolism pathways were activated in KIRC patients with a high expression of HGD or GSTZ1 (Figure 2A,B), which implies that alterations in tyrosine metabolism induced by HGD and GSTZ1 lead to global fluctuations in amino acid metabolism. By overlapping the analysis of amino acid metabolism-related genes affected by HGD and GSTZ1, we found that HGD and GSTZ1 jointly affect phenylalanine, tyrosine, glycine, serine, threonine, alanine, aspartate, glutamate, arginine, proline, and glutathione metabolism (Figure 2C,D).

To screen out the target genes regulated by HGD and GSTZ1, we performed an overlap analysis and found that glutamic-oxaloacetic transaminase (GOT1 and GOT2) were identified as potential target (Figure 2E). Similarly, when we performed high-low group analysis with GOT1 or GOT2 as the target gene, amino acid metabolism pathways were also enriched, indicating that GOT1 and GOT2 were involved in amino acid metabolism. More importantly, the expression of GOT1 and GOT2 was significantly decreased and were positively correlated with the expressions of HGD and GSTZ1 in KIRC (Figure 2H,I). These results suggest that GOT1 and GOT2 mediate the regulation of amino acid metabolism by HGD and GSTZ1 in KIRC.

### 2.3. HGD and GSTZ1 Promoted the Conversion of Glucose to Lactate in KIRC

**Figure 3:**
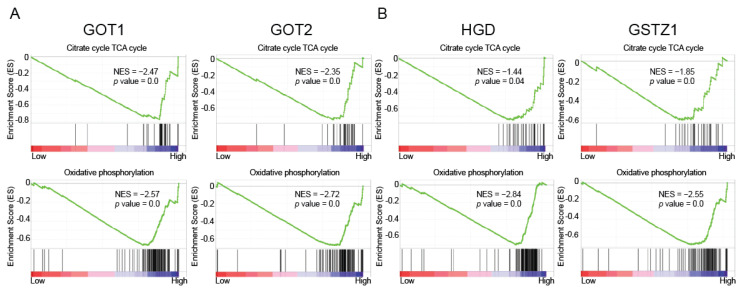

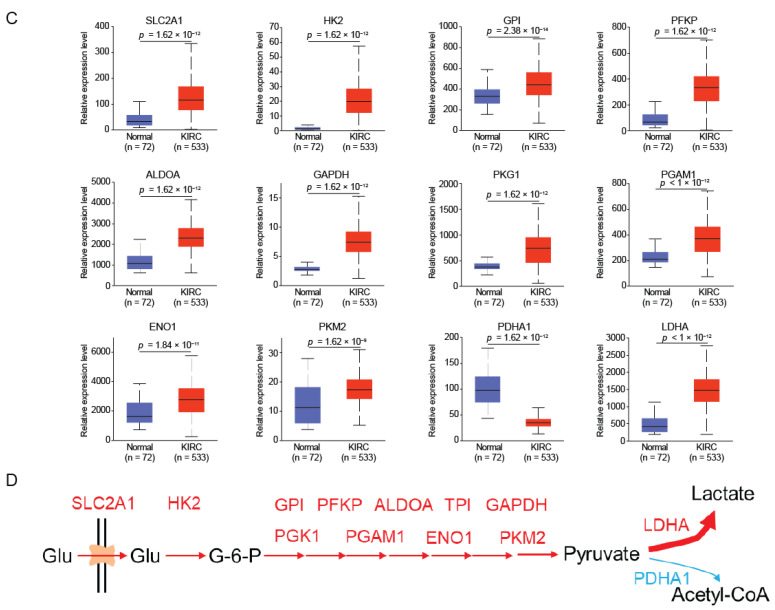
HGD/GSTZ1 promotes the conversion of glucose to lactate in KIRC. (**A**,**B**) GSEA pathway enrichment analyses of the key regulator (GOT1, GOT2, HGD, and GSTZ1) signature in patients with RCC from the TCGA datasets. (**C**) The glucose-metabolizing enzymes mRNA expression levels between tumor and normal tissues in patients with KIRC in TGCA database. (**D**) Schematic diagram of the glucose metabolism pathway.

GOT1 and GOT2 reversibly catalyzes the inter-conversion of aspartate and oxaloacetate (OAA), and thus coordinates the carbohydrate and amino acid metabolism. In mitochondria, oxidation of pyruvate by pyruvate dehydrogenase (PDH) generates acetyl coenzyme A (acetyl-CoA), which then combines with oxaloacetate (OAA) to form citrate, the first substrate of the TCA cycle. To explore the mechanism regulated for the energy metabolism by GOT1 and GOT2, we first examined the involvement of the oxidative phosphorylation and TCA cycle, and found that high expression of GOT1 and GOT2 promotes these metabolic processes (Figure 3A). Interestingly, a high expression of HGD and GSTZ1 also promoted these metabolic processes (Figure 3B), which suggests that HGD and GSTZ1 may regulate energy metabolism through GOT1 and GOT2.

However, the expression of these metabolic enzymes (HGD, GSTZ1, GOT1, and GOT2) is reduced in KIRC, which means that the oxidative phosphorylation and TCA cycle is inhibited and is not conducive to cell growth. Intracellular energy is mainly generated from extracellular uptake glucose, and we analyzed the expression levels of glucose metabolizing enzymes. The results showed that renal cancer cells increased the glucose uptake and production of pyruvate (Figure 3C), which is also in line with previous reports [8]. Pyruvate can be converted into either acetyl-CoA into the TCA cycle or into lactate. We found that PDHA1 expression was decreased, while lactate dehydrogenase (LDHA) expression was increased (Figure 3C). This suggests that renal cancer cells are more inclined to convert glucose to lactate rather than acetyl-CoA. Therefore, we found that the HGD/GSTZ1-GOT1/GOT2 axis drives renal cancer cells to undergo aerobic glycolysis, converting uptake glucose into lactate to obtain energy to meet their own needs (Figure 3D).

### 2.4. HGD/GSTZ1 Promoted Cell Cycle and Tumor Progression

**Figure 4:**
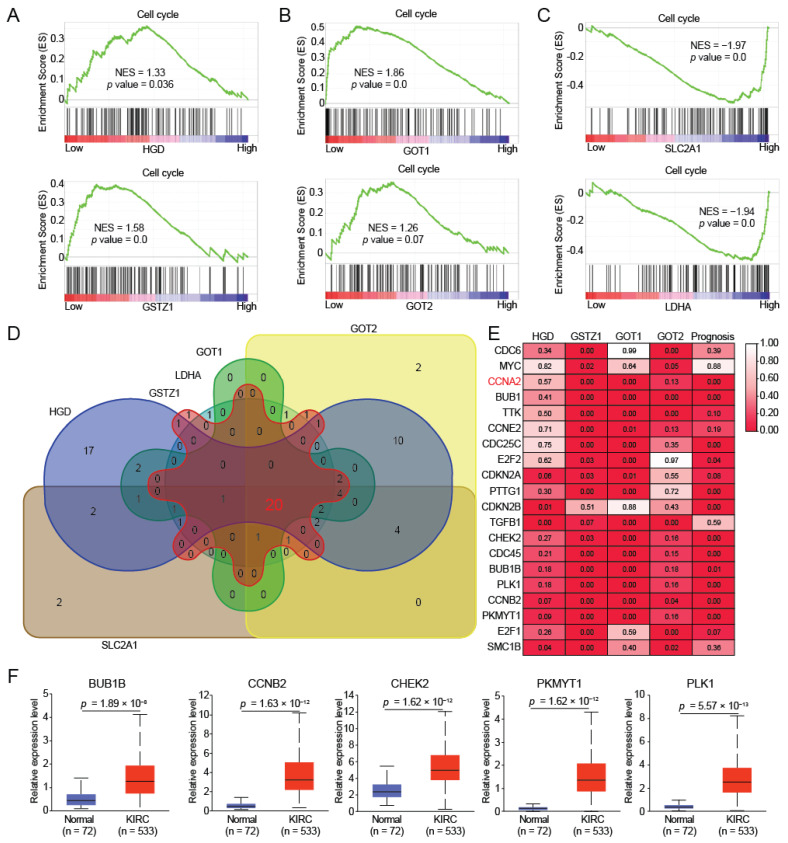
HGD/GSTZ1 promotes cell cycle and tumor progression. (**A**–**C**) GSEA pathway enrichment analyses of key regulator (HGD, GSTZ1, GOT1, GOT2, SLC2A1, and LDHA) signature in patients with RCC from the TCGA datasets. (**D**) The overlapping analysis for related genes of cell cycle in RCC patients. The number represents the number of genes shared by different gene sets. (**E**) The correlation analysis between HGD/GSTZ1/GOT1/GOT2/prognosis and cell cycle related target genes. (**F**) The cell cycle related key target genes mRNA expression levels between tumor and normal tissues in patients with KIRC in TGCA database.

The energy produced by tumor cells is mainly used to promote cell proliferation. Our previous work found that decreased tyrosine metabolism promotes cell cycle in hepatocellular carcinoma [12], and we found that down-regulation of HGD and GSTZ1 also promotes cell cycle in KIRC (Figure 4A). In addition, GOT1 and GOT2, which have a positive correlation with HGD and GSTZ1 expression, also promoted cell cycle at low expression (Figure 4B); on the contrary, SLC2A1 and LDHA with high expression promoted cell cycle (Figure 4C). These results suggest that HGD and GSTZ1 regulate the cell cycle of renal cancer cells through GOT1, GOT2, SLC2A1, and LDHA.

To further determine the regulation of cell cycle by HGD and GSTZ1, we screened out 20 cell cycle-related target genes (Figure 4D). Furthermore, we also analyzed the expression correlation between cell cycle-related target genes and HGD, GSTZ, GOT1, GOT2, and found a significant expression correlation (Figure 4E). More importantly, their expression was also closely linked to the prognosis of KIRC (Figure 4E). Among them, we selected BUB1B, CCNB2, CHEK2, PKMYT1, and PLK1, and found that their expressions were all significantly elevated in KIRC (Figure 4F).

**Figure 5:**
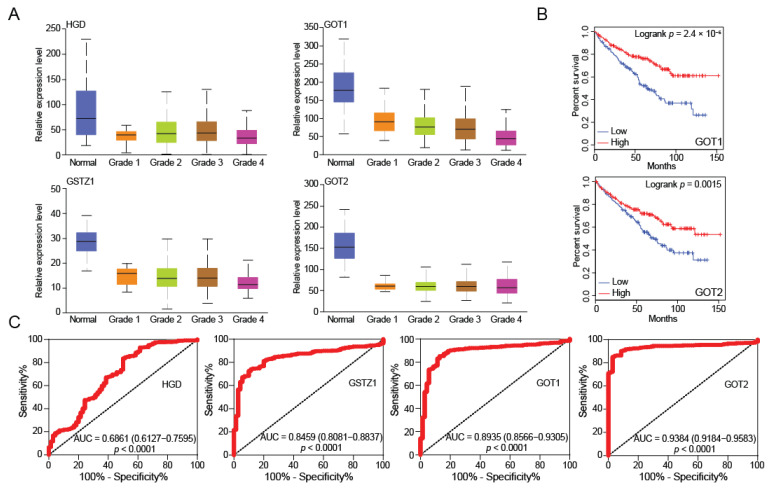

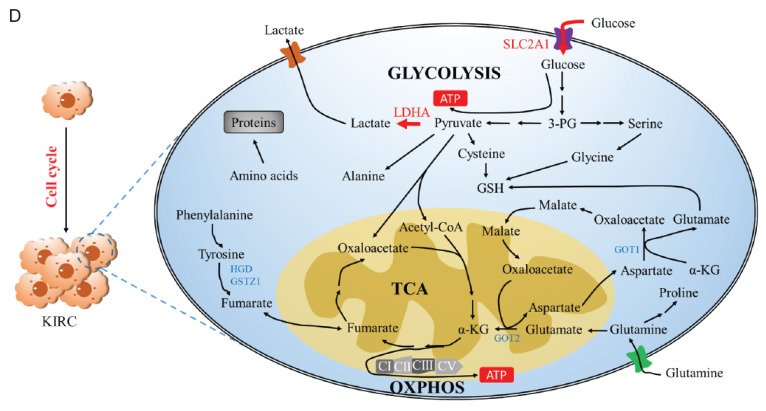
Down-regulation of HGD and GSTZ1 may serve as a potential diagnostic biomarker in KIRC. (**A**) The key regulator (HGD, GSTZ1, GOT1, and GOT2) mRNA expression levels between different grades of tumor and normal tissues in patients with KIRC in TGCA database. (**B**) Overall survival of patients with KIRC grouped by GOT1 and GOT2 expression through GEPIA web server. (**C**) ROC curve analysis indicated that the key regulator (HGD, GSTZ1, GOT1, and GOT2) could efficiently distinguish KIRC from normal individual. AUC, the area under curve. (**D**) Proposed model: The role and mechanism of HGD and GSTZ1 in KIRC progression.

In addition, we also found that the expression of HGD, GSTZ1, GOT1, and GOT2 gradually decreased as the grade of KIRC increased (Figure 5A), which suggests that their expression level indicates the degree of KIRC progression. More importantly, a low expression of GOT1/2 in tumor tissues are significantly associated with poor OS in patients with KIRC (Figure 5B). To probe whether the low expression of HGD and GSTZ1 possesses diagnostic significance in KIRC patients, the ROC curves were used to analyze the diagnostic value of HGD and GSTZ1 expression from TCGA-KIRC datasets. ROC curve analysis showed that HGD and GSTZ1 could statistically distinguish KIRC from normal tissue, producing an area under the curve (AUC) of 0.6861 (95% CI: 0.6127–0.7595; *p* < 0.0001) and 0.8459 (95% CI: 0.8081–0.8837; *p* < 0.0001), respectively (Figure 5C). Moreover, we performed a ROC curve analysis against GOT1 and GOT2 expression. The ROC curve analysis implied that the low expression of GOT1/GOT2 might have diagnostic value for KIRC patients (Figure 5C). These results implicitly suggested that the key regulator (HGD, GSTZ1, GOT1, and GOT2) might have diagnostic value for patients with KIRC. In this study, we have found that HGD and GSTZ1 regulate the level of fumarate by metabolizing tyrosine, and by affecting GOT1 and GOT2 to coordinate amino acid metabolism and energy metabolism, determine the energy production pathway of tumor cells, and ultimately regulate the cell cycle (Figure 5D). We believe that the tyrosine metabolizing enzymes HGD and GSTZ1 are reliable biomarkers of KIRC, which will provide important help for the clinical diagnosis and treatment of renal cancer.

## 3. Discussion

Renal cell carcinoma is the most common tumor in the urinary system. Early diagnosis and timely surgical treatment are key factors in the treatment of localized RCC. However, due to the lack of reliable and specific diagnostic biomarkers, many patients have advanced stage and progressed into metastasis at clinical diagnosis, resulting in poor prognosis. Therefore, early screening of these patients is beneficial to the treatment and prognosis. However, there is currently no effective biomarker for early diagnosis of RCC clinically.

The uncontrolled proliferation of cancer cells involves not only deregulated control of cell proliferation but also corresponding adjustments of energy metabolism in order to fuel cell growth and division. To ensure rapid proliferation, tumor cells reshape the flow of metabolism. It is known that tumor cells preferentially convert glucose into lactate for energy, which is known as aerobic glycolysis. This reprogramming of energy metabolism is seemingly irrational, as cancer cells must compensate for the lower efficiency of ATP production afforded by glycolysis relative to oxidative phosphorylation. In our results, however, we did find that the expression of glucose transporter GLUT1 (SLC2A1 encoded protein) is increased, suggesting an increased glucose uptake. Interestingly, the TCA cycle and oxidative phosphorylation were also inhibited. Meanwhile, the expression of lactate dehydrogenase (LDHA), converting pyruvate to lactate, was also increased, indicating that the majority of uptake glucose was converted to lactate. These results are in line with previous reports of increased glucose uptake, suppressed TCA cycle, and dysregulated amino acid metabolism [8,9]. More importantly, we also found significantly lower levels of fumarate in renal cancer [10]. Fumarate is an important metabolite in the TCA cycle, but its levels cannot significantly alter the TCA cycle. Therefore, fumarate may be regulated by other metabolic pathways. In our previous study, it was found that tyrosine metabolism plays an important tumor suppressor role in hepatocellular carcinoma, and its end product includes fumarate [12]. We found that tyrosine metabolizing enzymes HGD and GSTZ1 were significantly decreased in KIRC, which suggests that the reduction in fumarate may be due to decreased tyrosine metabolism. After an in-depth analysis, we found that HGD and GSTZ1 not only regulate the TCA cycle and oxidative phosphorylation, but also alter amino acid metabolism and glucose metabolism. These results suggest that metabolic processes in RCC are interconnected, and amino acid metabolism links energy metabolism and glucose metabolism through intermediate metabolites, which enriches the context for tumor metabolic reprogramming.

HGD and GSTZ1 are metabolic enzymes in tyrosine metabolism and play important functions in amino acid metabolism. However, there is not much research on tyrosine metabolism. The research on HGD has mainly focused on alkaptonuria, and it is believed that HGD and alkaptonuria are closely related [18,19,20,21]. On the other hand, GSTZ1 is considered to be a tumor suppressor in hepatocellular carcinoma cells [12,22,23,24,25]. Importantly, no articles have reported HGD and GSTZ1 as potential biomarkers of KIRC, so our study is the first to identify HGD and GSTZ1 as tumor biomarkers, which fills the gap of tyrosine metabolism in the field of tumor markers.

The kidney is an important organ in the human body. Its main function is to generate urine to excrete wastes from the body, and at the same time to reabsorb useful substances, including glucose and protein, which indicates that active metabolic events are always taking place in kidney tissue. Metabolic reprogramming is a distinct feature of kidney cancer and is therefore suitable for identifying biomarkers. In our study, we found that the low expression of tyrosine metabolizing enzymes HGD and GSTZ1 was significantly associated with poor prognosis in KIRC, and their expression decreased with the increasing RCC grade. They not only coordinated energy metabolism by regulating the expression of GOT1 and GOT2, but also altered glucose metabolism by regulating the expression of SLC2A1 and LDHA1, which made HGD and GSTZ1 reliable biomarkers for RCC.

## 4. Materials and Methods

### 4.1. The Analysis for Differential Gene Expression and Mutation

The mRNA expression and protein expression data were obtained from The Cancer Genome Atlas (TCGA: http://ualcan.path.uab.edu/analysis (accessed on 18 March 2022)) [26] and Clinical Proteomic Tumor Analysis Consortium (CPTAC: http://ualcan.path.uab.edu/analysis-prot (accessed on 18 March 2022)) [27]. The somatic mutation data was obtained from TCGA cBioportal platform (https://www.cbioportal.org/ (accessed on 18 March 2022)) [28,29]. The mutation was identified and analyzed in the cBioportal platform.

### 4.2. Survival Prognostic Analysis

The patient survival analysis was run in GEPIA web server (http://gepia.cancer-pku.cn/detail.php?gene=&clicktag=survival (accessed on 19 March 2022)) [30]. GEPIA is a newly developed interactive web server for analyzing the RNA sequencing expression data of 9736 tumors and 8587 normal samples from the TCGA and the GTEx projects, using a standard processing pipeline. GEPIA provides customizable functions such as tumor/normal differential expression analysis, profiling according to cancer types or pathological stages, patient survival analysis, similar gene detection, correlation analysis, and dimensionality reduction analysis.

### 4.3. Gene Set Enrichment Analysis (GSEA)

Gene set enrichment analysis was performed using GSEA 4.0.3 (Broad Institute, Cambridge, MA, USA) (http://software.broadinstitute.org/gsea/index.jsp (accessed on 19 March 2022)) in which the hallmark gene set “c2.cp.kegg.v7.5.symbols.gmt” was adopted. For the grouping of patients, we ranked the specific gene expression in order from high to low, and divided the number of patients in half. The top-ranked patients were divided into the high-expression group, and the remaining patients were divided into the low-expression group.

### 4.4. Correlation Analysis

Expression of the indicated genes was obtained from the TCGA database, and correlation analysis was performed using GraphPad Prism 8 (GraphPad Software Inc., San Diego, CA, USA). A Pearson coefficient (r value) greater than zero represents a positive correlation, and less than zero represents a negative correlation. The larger the absolute value of r, the stronger the correlation.

### 4.5. Target Overlap Analysis

The overlap analysis of target genes was performed by “Calculate and draw custom Venn diagrams” (http://bioinformatics.psb.ugent.be/webtools/Venn/ (accessed on 19 March 2022)), which is provided as “free to use for all”.

### 4.6. Statistics

Data were analyzed using GraphPad Prism 8 (GraphPad Software Inc., San Diego, CA, USA). All data are presented as mean ± standard deviation. Comparison of two groups was conducted using the two-tailed Student’s *t*-test. A value of *p* < 0.05 was considered to indicate a statistically significant difference.

## 5. Conclusions

In summary, we confirm that the low expression of HGD and GSTZ1 promoted the progression and poor prognosis in KIRC. Mechanistically, we found that decreased HGD and GSTZ1 regulate amino acid metabolism to reduce fumarate production, which in turn remodels metabolic flux and energy production in renal cancer cells, ultimately promoting cell cycle and proliferation. Furthermore, these results suggest that HGD and GSTZ1 have the potential not only to be promising biomarkers for the diagnosis and prognosis of KIRC patients, but also to provide new directions and strategies for KIRC treatment. More importantly, this is the first report of the amino acid metabolizing enzymes HGD and GSTZ1 as tumor biomarkers. However, it is undeniable that these KIRC related differential driving genes need further experimental verification on the basis of a rigorous attitude.

## Data Availability

All data will be made available upon reasonable request by emailing the corresponding author.
